# The Differential Impact of Emotional Support on Social Problem‐Solving and Mental Health Among Latina Immigrant Survivors of Adverse Childhood Experiences

**DOI:** 10.1002/nur.70038

**Published:** 2025-12-09

**Authors:** Laura Mata Lopez, Owen Smith, Maria Jose Sanchez‐Roman, Lia Escobar‐Acosta, Carmen Alvarez

**Affiliations:** ^1^ School of Nursing Johns Hopkins University Baltimore Maryland USA; ^2^ College of Public Health University of Nebraska Medical Center Omaha Nebraska USA; ^3^ University of Pennsylvania School of Nursing Philadelphia Pennsylvania USA

**Keywords:** anxiety, CLINICAL TOPICS, CLINICAL TOPICS, depression, CLINICAL TOPICS, psychological attributes, CLINICAL TOPICS, social support, emotional states/feelings, health promotion/weIlness behaviors

## Abstract

Adverse childhood experiences (ACEs) are often positively associated with mental health conditions. We examined whether emotional support attenuates the association between ACE clusters and both social problem solving and mental health assessments (depression, anxiety, post‐traumatic stress disorder (PTSD) symptoms). A convenience sample of 336 Latina immigrant women completed questionnaires about their ACEs, emotional support, social problem‐solving, and depression, anxiety, and PTSD symptoms. Using PROCESS SPSS Macro version 4.0 we conducted four separate moderation analyses. We identified 5 ACEs clusters from our sample (*n* = 336): Global ACEs (15.5%), Community Violence and Physical Abuse (23.8%), Physical and Emotional Abuse (21.4%), Household Dysfunction with Physical and Emotional Abuse (16.7%), and Low ACEs (22.6%). Emotional support served as a protective moderator with differential effects, providing the greatest benefit to women who experienced high levels of abuse. For social problem‐solving abilities, significant interactions emerged for the Physical and Emotional Abuse and Household Dysfunction clusters, indicating that as emotional support increases, the difference in social problem‐solving between these high‐ACE clusters and the Low ACEs cluster decreased. For PTSD symptoms, women in the Global ACEs cluster showed significantly higher symptoms than the Low ACEs group when emotional support was low. The clusters did not differ at high emotional support levels, indicating that adequate social support can reduce the negative effects of ACEs on both PTSD symptoms and effective problem‐solving. These findings underscore the need for trauma‐informed, culturally responsive care models that integrate ACE screening and guide future interventions to strengthen social support networks for ACE survivors.

Mental health disparities disproportionately affect Latinos in the United States, including immigrant Latinos who face increased risk for elevated depression and anxiety symptoms (Kochanek et al. 2020; Ryan et al. 2021; Substance Abuse and Mental Health Services Administration SAMSHA 2024). A notable risk factor for poor mental health outcomes in adulthood are adverse childhood experience (ACEs), which are stressful and potentially traumatic events that occur in childhood, including abuse, neglect, family dysfunction, or witnessing violence (Centers for Disease Control and Prevention 2025). ACEs are also linked to maladaptive problem‐solving styles which further contribute to mental health challenges (Merrick et al. 2017; Stubbs et al. 2017; Ryan et al. 2021). Although the link between ACEs and mental health has been documented among Latinos (Alvarez et al. 2019, 2022), less research has explored protective factors, such as emotional support, that may mitigate these negative effects. Importantly, existing literature suggests that Latina women report higher rates of both ACEs and mental health symptoms compared to Latino men (Llabre et al. 2017). This study therefore investigates whether emotional support moderates the relationships between ACEs and both social problem‐solving skills and mental health outcomes within this vulnerable population.

## 1 Compounding Stressors for Latina Immigrants

The recent influx of Latina immigrants into the United States has been driven, in part, by sociopolitical instability in Central and South America (Ward and Batalova 2023). Before and during migration, many Latina migrants experience extreme poverty, political oppression, and gang violence (Vargas et al. 2024). Once in the United States, acculturation difficulties, unstable and low‐paying jobs, and limited access to healthcare can further exacerbate their mental health struggles (Rios Casas et al. 2020; Ryan et al. 2021; Valentín‐Cortés et al. 2020).

Concurrent with migration and associated hardships, many Latina immigrants experience significant mental health challenges, with approximately one‐third experiencing clinically‐significant depression or anxiety (Alvarez et al. 2022). This prevalence is particularly high among those with a history of adverse childhood experiences (ACEs) (LaBrenz et al. 2020; Llabre et al. 2017). Critically, Latina immigrants report disproportionately high rates of ACEs (78%) compared to Latino immigrant men (31.2%) (LaBrenz et al. 2020; Llabre et al. 2017). This disparity contributes to more frequent and chronic depressive episodes, often resulting in more challenging treatment outcomes (Gloger et al. 2021; Negele et al. 2015; Rios Casas et al. 2020; Ryan et al. 2021; Valentín‐Cortés et al. 2020; Vargas et al. 2024). This accumulation of risk factors across the lifespan makes Latina immigrant women particularly vulnerable to psychological distress and an important population for understanding how protective factors like emotional support may buffer against these multilayered stressors.

## 2 Social Problem‐Solving and Adverse Childhood Experiences

Social problem‐solving refers to the cognitive‐behavioral process individuals utilize to identify and implement effective strategies for managing daily stressors and challenges (D'Zurilla and Chang 1995; D'Zurilla and Goldfried 1971). Effective problem‐solving styles, characterized by positive problem orientation and rational approaches, are negatively associated with psychological distress and promote positive well‐being (Nezu et al. 2004). Conversely, ineffective strategies, such as negative problem orientation, avoidance, and impulsivity, are linked to increased psychological distress and hinder overall well‐being. ACEs negatively impact cognitive development (Dube et al. 2004) as well as self‐efficacy, adaptive coping strategies, and problem‐solving styles (Futa et al. 2003; Hager and Runtz 2012; Merrick et al. 2017). Studies have shown that ACE survivors, particularly those with a greater number of abuse experiences, exhibit lower overall social problem‐solving skills, a more negative problem orientation, and a stronger reliance on avoidance behaviors compared to individuals with fewer ACEs (Alvarez et al. 2022).

Latina immigrant ACE survivors, particularly those who experienced community violence and physical abuse, benefit significantly from strong social problem‐solving skills. Researchers (Alvarez et al. 2022) identified a link between higher social problem‐solving skills and both greater life satisfaction and reduced depressive, anxiety, and PTSD symptoms among Latina immigrants. Results also indicated that social problem‐solving may play a differential role in the pathway between ACEs and mental health. Specifically, for women who reported community violence and physical abuse ACEs, higher social problem‐solving skills appeared to be protective, buffering the negative impact of specific ACEs on depressive and anxiety symptoms. These findings highlight the importance of discerning nuanced relationships beyond the cumulative burden of ACEs. Understanding how protective factors (like social problem‐solving and emotional support) affect people who have experienced different types of early life adversity is crucial for promoting positive mental health outcomes among Latina immigrants.

## 3 Emotional Support Buffers the Impact of Stress

Decades of research (Cohen 2004; Folkman et al. 1986; Heaney and Israel 2008) support the buffering effect of emotional support, where having someone to confide in during challenging times fosters a sense of security, improves emotional regulation, and bolsters self‐efficacy or the belief in your ability to cope. This is particularly true for ACE survivors. Studies (Brinker and Cheruvu 2017; Ryan et al. 2021; Cheong et al. 2017) demonstrate that emotional support significantly reduces the likelihood of experiencing depressive symptoms. While broader social networks offer Latino(a) immigrants protection from migration‐related stress (Garcini et al. 2021; Ornelas et al. 2020) emotional support fosters a deeper sense of connection and empowers individuals by increasing their knowledge of personal and community resources (Crocker 2015; Rios Casas et al. 2020; Ryan et al. 2021). However, the immigration process itself often disrupts these vital support systems, leading to isolation, psychological distress, and unhealthy coping mechanisms (Crocker 2015; Falconier et al. 2016; Rios Casas et al. 2020). This social network disruption occurs precisely when women face new stressors requiring adaptive coping and resilience. Understanding how available emotional support can buffer the relationship between past ACEs and current mental health symptoms is therefore essential for this population, as it can inform interventions that either strengthen remaining support networks or help rebuild social connections in their new environment. Given that Latina immigrant women face both heightened psychosocial stressors and reduced access to traditional sources of emotional support, identifying the protective potential of available support becomes crucial for promoting resilience and preventing mental health deterioration in this at‐risk population.

## 4 Nursing and Healthcare Worker Relevance

As healthcare systems increasingly implement routine screening for ACEs and awareness grows regarding their profound impact on lifelong health outcomes, nurses find themselves at the forefront of both identifying trauma exposure and responding to its consequences (Srivastav et al. 2021; Shimkhada et al. 2022). Nurses, by the very nature of their training and practice, are uniquely positioned to address the complex interplay between social determinants of health and mental well‐being among vulnerable populations. The holistic approach of nursing care extends beyond acute care to encompass health promotion, disease prevention, and community‐based interventions—competencies that are fundamental when working with underserved Latina immigrant survivors of ACEs. As often the first point of contact across diverse healthcare settings—from community clinics and schools to hospitals—nurses and other healthcare workers are strategically positioned to not only screen for and identify childhood trauma exposure but also to recognize and foster the protective factors that can mitigate its long‐term effects.

## 5 Current Study

This study goes beyond examining the overall association between cumulative ACEs and mental health by examining whether emotional support attenuates the negative association between specific types of ACEs and both social problem‐solving styles and mental health symptoms (depression, anxiety, and PTSD) in Latina immigrants. Despite this strategic positioning and the growing emphasis on trauma‐informed care, the literature currently lacks research on how emotional support impacts the mental health of Latina immigrant ACE survivors. This study aims to fill this gap by investigating how emotional support can enhance the psychological well‐being of this population, potentially mitigating the psychological effects of childhood trauma. This exploration might reveal promising intervention targets and inform healthcare practices for Latina immigrants who have experienced ACEs.

## 6 Methods

### 6.1 Sample and Data Collection

We recruited a convenience sample of Latina immigrant women (*n* = 336) recruited between April to July 2019 from primary care settings. A bilingual research assistant approached potential participants and explained the study on women's health and stress. To be eligible, women had to identify as Latina, be born outside the United States (including Puerto Rico), and report at least one adverse childhood experience. Those who met the criteria and were willing to be in the study provided informed consent and completed a questionnaire in English or Spanish. A research assistant administered the survey with participants in‐person and entered responses into a secure online platform (REDCap). All participants received $20 compensation for their time. The study was approved by both the University and Clinic Institutional Review Boards.

### 6.2 Measures

#### 6.2.1 Dependent Variables

##### 6.2.1.1 Social Problem‐Solving

Participants' social problem‐solving styles were assessed using the Social Problem‐Solving Inventory‐Revised (SPSI‐R; D'Zurilla and Chang 1995). This 25‐item self‐report measure evaluates problem orientation (positive or negative) and problem‐solving styles (rational, impulsive/careless, and avoidant) using a 5‐point Likert scale (0 = “Not at all true of me” to 4 = “Extremely true of me”). Higher total scores indicate better social problem‐solving skills. The SPSI‐R has demonstrated good reliability and validity in previous research (De La Torre et al. 2010; Vázquez et al. 2016). In this study, internal consistency was adequate (α = 0.74).

##### 6.2.1.2 Depression Symptoms

Depressive symptoms were assessed using the eight‐item Patient Health Questionnaire (PHQ‐8), a widely recognized screening tool (Wu et al. 2020). Participants indicated the frequency of experiencing each symptom on a 4‐point Likert scale (0 = “not at all” to 3 = “nearly every day”). A total symptom score was calculated, with higher scores reflecting greater depressive symptomatology. The PHQ‐8 has demonstrated strong psychometric properties in diverse populations, including Spanish‐speaking communities (Alpizar et al. 2018; Pagán‐Torres et al. 2020). In the present study, the PHQ‐8 exhibited good internal consistency reliability (α = 0.83).

##### 6.2.1.3 Anxiety Symptoms

To measure anxiety symptoms, we utilized the Generalized Anxiety Disorder 7‐item (GAD‐7) scale developed by Spitzer et al. (2006). This self‐report measure asks participants to indicate how often they have experienced seven common anxiety‐related symptoms over the past 2 weeks. Response options range from “not at all” (0) to “nearly every day” (3). Individual item scores are summed to create a total score, with higher scores reflecting greater severity of anxiety symptoms. The GAD‐7 has been extensively validated and has shown good psychometric properties in diverse samples, including Spanish‐speaking communities (Wong et al. 2010; Mills et al. 2014). Consistent with previous findings, the GAD‐7 exhibited good internal consistency in our study (α = 0.88).

##### 6.2.1.4 Post‐Traumatic Stress Disorder Symptoms

PTSD symptoms were evaluated with the 5‐item Primary Care PTSD Screen for DSM‐5 (PC‐PTSD‐5), developed by Prins et al. (2016). This brief measure asks respondents to indicate whether they have experienced specific PTSD symptoms in the past month, including nightmares, hypervigilance, avoidance of reminders, emotional numbing, and persistent feelings of guilt. Each item is scored as “yes” (1) or “no” (0), with a higher total score indicating greater PTSD symptom severity. The PC‐PTSD‐5 demonstrated good internal consistency in this study (α = 0.70).

#### 6.2.2 Moderator Variable

##### 6.2.2.1 Emotional Support

To assess participants' perceptions of emotional support, we used the 6‐item PROMIS Emotional Support scale (Hahn et al. 2010). This measure evaluates the frequency with which individuals experience feelings of being valued and cared for by others, as well as having people they can rely on for support and confide in about their problems. Example items include, “I have someone to confide in or talk to about myself or my problems” and “I have someone who understands my problems.” Responses are on a 5‐point Likert scale ranging from 1 (never) to 5 (always). A higher total score on the measure reflects greater perceived emotional support. In this study, the PROMIS Emotional Support item bank showed good internal consistency (α = 0.93).

#### 6.2.3 Independent Variable

##### 6.2.3.1 Adverse Childhood Experiences

Childhood adversity was assessed with the 36‐item Adverse Childhood Experiences International Questionnaire (World Health Organization 2018). This measure asks about various forms of maltreatment and exposure to toxic stress before age 18, including abuse, neglect, household dysfunction, peer and community violence, and collective violence (e.g., “Were you beaten up by soldiers, police, militia, or gangs?”). Although the original response options referred to the frequency of experiences, responses were dichotomized (yes/no) for this study.

#### 6.2.4 Covariates

In addition to demographic information (age, education, employment, and relationship status), we assessed chronic life burden using the 15‐item Chronic Burden Scale (CBS) (Gurung et al. 2004). Developed for minoritized and underserved women, the CBS measures the impact of difficulties fulfilling social roles due to chronic stressors (e.g., financial, legal, medical). Participants rated the impact of these stressors over the past 6 months (1 = not a problem to 4 = major problem), with higher scores indicating greater burden. This measure, previously validated in a diverse community sample including Latina women (Gurung et al. 2004), demonstrated good internal consistency in the current study (α = 0.76).

#### 6.2.5 Data Analysis

We conducted all analyses using IBM SPSS Statistics. We reported the development of the ACE clusters using hierarchical cluster analysis as described elsewhere (Alvarez et al. 2022). To summarize, hierarchical cluster analysis with Ward's method and squared Euclidean distance was employed to identify groups of women with similar adverse childhood experience (ACE) profiles based on their dichotomized (yes/no) responses to the ACE‐IQ. The optimal number of clusters was determined using the agglomeration schedule. To validate the resulting cluster solution, k‐means cluster analysis was conducted. Chi‐square tests and ANOVAs were then performed to examine differences between the ACE clusters in terms of problem‐solving styles, chronic life burden, and indicators of psychosocial well‐being. Next, we used the PROCESS SPSS macro version 4.2 to test our moderation hypotheses (Hayes 2012). We examined whether emotional support moderated (1) the associations between the ACEs clusters and social problem‐solving, and (2) the direct associations between ACEs clusters and the mental health variables. We conducted separate moderation analyses for each dependent variable, in which we treated the five clusters as independent variables (using the Low ACEs cluster as the reference), indicated emotional support as the moderator, and specified social problem‐solving and the mental health indicators (depression, anxiety, PTSD symptoms) as the dependent variables. The covariates were included in the moderation models. We determined a priori that a sample size of 300 will allow us to detect small effect size of 0.26 in our regression analyses with statistical power of 0.80 and alpha of 0.05. Missing data analysis revealed less than 5% missing values across study variables. Cases with missing data on study variables were excluded using listwise deletion.

##### 6.2.5.1 Post‐Hoc Mediation Analyses

Given that emotional support did not significantly moderate the relationships between ACE clusters and depression or anxiety symptoms, we conducted exploratory post‐hoc mediation analyses to examine whether emotional support might instead function as a mediator in these relationships. Mediation analyses were performed using Hayes' PROCESS macro version 4.2 (Model 4), which employs ordinary least squares regression‐based path analysis to estimate both direct and indirect effects. Mediation was considered present when the 95% confidence interval for the indirect effect excluded zero. The covariates were included in the mediation models.

## 7 Results

### 7.1 Sample Characteristics

The demographic characteristics of our sample are presented in Table 1. Overall, most of our participants (*n* = 336) were married/cohabitating (71.7%), had less than a high school education (39.3%), were unemployed (61.6%), and emigrated from Central America: El Salvador (24.3%), Guatemala (11%), Honduras (34.4%). Participants from Mexico (18.2%) and the South American and Caribbean region (12.1%) were also represented in the sample. At the time of data collection, participants had been living in the United States for an average of 8.6 years, and level of acculturation was low (*M* = 1.4; SD = 0.83).

**TABLE 1:** Demographics of participants.

Characteristics	%	*n*	*M*	SD
Total sample	100	336		
Age			31.5	7.95
Relationship status				
Married/cohabitating	71.7	241		
Single/divorced	28.3	95		
Education				
Elementary school or less	39.3	132		
Some high school education	26.8	90		
High school graduate or more	33.9	114		
Job status				
Not employed	61.6	207		
Employed	38.4	129		
Place of birth				
Mexico	18.2	63		
El Salvador	24.3	84		
Guatemala	11.0	38		
Honduras	34.4	119		
South America and Caribbean	12.1	42		
Number of years in United States			8.6	5.98
Level of acculturation			1.35	0.83
Moderator and outcome variables				
Social problem‐solving			14.0	2.57
Emotional support			25.3	6.93
Depression			6.7	5.88
Anxiety			6.4	6.22
Post‐traumatic stress symptoms			1.73	1.56

### 7.2 ACEs Clusters

We identified five clusters of ACEs in this community sample (Low ACEs, Physical and Emotional Abuse, Household Dysfunction with Physical and Emotional Abuse, Community Violence and Physical Abuse, and Global ACEs). Cluster nomenclature was derived from the most prevalent or absent ACEs within each group. The smallest cluster, Global ACEs, was characterized by a high likelihood of exposure to a broad spectrum of ACEs, including sexual, physical, and emotional abuse; household dysfunction; community and collective violence; and bullying. The Community Violence and Physical Abuse cluster was comprised of individuals predominantly reporting physical abuse and exposure to community and collective violence. Participants in the Physical and Emotional Abuse cluster primarily endorsed experiences of physical and emotional abuse. Similarly, the Household Dysfunction with Physical and Emotional Abuse cluster also reported physical and emotional abuse, along with household dysfunction such as witnessing abuse and parental substance misuse. Finally, the Low ACEs cluster represented individuals with a low probability of experiencing most ACEs. Our subsequent analysis, summarized in Table 2, highlights the differences in emotional support, social problem‐solving, and the prevalence of depression, anxiety, and PTSD symptoms among the distinct ACE clusters. There were statistically significant differences between all ACE clusters on social problem‐solving [*F* (4, 321) = 2.91, *p* = 0.022]; depression symptoms [*F* (4, 323) = 7.29, *p* < 0.001]; anxiety symptoms [*F* (4, 323) = 6.41, *p* < 0.001]; PTSD symptoms [*F* (4, 319) = 7.96, *p* < 0.001]; and emotional support [*F* (4,320) = 4.98, *p* = 0.001]. Compared to all other ACE clusters, the Global ACEs group had lower scores for social problem‐solving and emotional support (indicating lower levels of emotional support and social problem‐solving skills) and the highest mean scores for depression, anxiety, and PTSD symptoms.

**TABLE 2:** Emotional support, social problem‐solving, and depression, anxiety, and PTSD symptoms by ACE clusters.

	Global ACEs (*n* = 52, 15.5%)		Community Violence and Physical Abuse (*n* = 80, 23.8%)		Physical and Emotional Abuse (*n* = 72, 21.4%)		Household Dysfunction with Physical and Emotional Abuse (*n* = 56, 16.7%)		Low ACEs (*n* = 76, 22.6%)		
	*n*	%	*n*	%	*n*	%	*n*	%	*n*	%	*p*
Emotional support	22.2	8.69	25.6	6.60	26.5	5.46	24.4	6.85	27.3	6.29	0.00
Social problem‐solving	13.1	2.8	14.4	2.4	14.4	2.4	14.3	2.4	13.7	2.8	0.02
Depression	9.5	6.5	7.9	6.1	4.9	4.6	6.1	5.8	5.2	5.2	0.00
Anxiety	8.8	6.4	7.4	6.3	4.5	5.3	7.5	6.8	4.5	5.7	0.00
Post‐traumatic stress	2.6	1.7	2.0	1.7	1.4	1.2	1.7	1.5	1.2	1.3	0.00

### 7.3 Moderating Effects of Emotional Support

**FIGURE 1:**
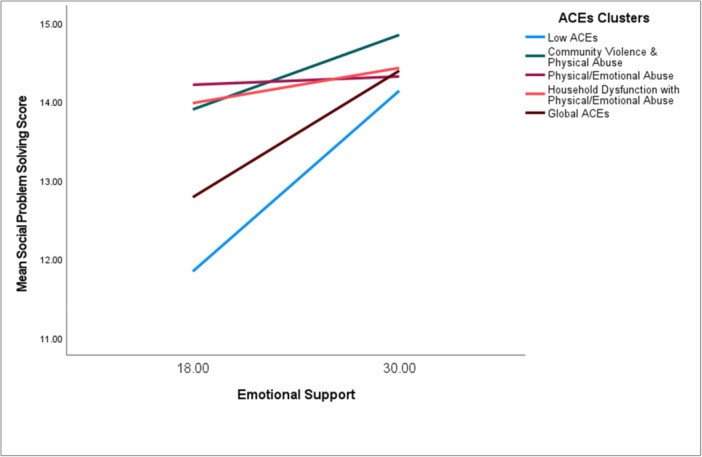
Social problem‐solving across ACE clusters by emotional support level among Latina immigrants. *Note:* Emotional support total scores range from 18 to 30, low to high, respectively.

Our multiple regression analyses showed that women in the Community Violence and Physical Abuse, Physical/Emotional Abuse, and Household Dysfunction with Physical/Emotional Abuse clusters reported higher social problem‐solving compared to women in the Low ACEs cluster (Table 3). Beyond the direct positive association between emotional support and social problem‐solving, moderation analyses were conducted to examine how emotional support influenced the relationship between ACE cluster membership and social problem‐solving. Results indicated a significant moderating effect of emotional support for women in the Physical and Emotional Abuse (*b* = −0.18, SE = 0.07, *t* = −2.58, *p* =0.010) and the Household Dysfunction with Physical/Emotional Abuse (*b* = −0.14, SE = 0.07, *t* = −2.28, *p* = 0.03) clusters. These findings suggest that at higher levels of emotional support, the negative association between belonging to these specific ACE clusters and social problem‐solving was significantly reduced, compared to the association between the Low ACEs cluster and social problem‐solving (Figure 1).

**TABLE 3:** Moderating role of emotional support in the association between ACE clusters and social problem‐solving, and ACE clusters and PTSD symptoms.

	Social Problem Solving				PTSD Symptoms			
	*β*	*SE*	95% CI	*p*	*β*	*SE*	95% CI	*p*
Low ACEs (reference)								
Community Violence and Physical Abuse (C1)	**4.06**	**1.70**	**[0.71, 7.40]**	**0.01**	**1.92**	**0.98**	**[−0.00, 3.84]**	**0.05**
Physical/Emotional Abuse (C2)	**5.55**	**1.92**	**[1.76, 9.34]**	**0.00**	0.52	1.10	[−1.64, 2.70]	0.63
Household Dysfunction with Physical/Emotional Abuse (C3)	**4.65**	**1.80**	**[1.12, 8.18]**	**0.00**	1.66	1.02	[−0.35, 3.69]	0.10
Global ACEs (C4)	1.80	1.62	[−1.39, 5.00]	0.26	**2.70**	**0.93**	**[0.87, 4.54]**	**0.00**
Emotional Support	**0.18**	**0.04**	**[0.09, 0.28]**	0.00	0.01	0.02	[−0.04, 0.06]	0.68
C1 * Emotional Support	−0.11	0.06	[−0.24, 0.00]	0.06	−0.05	0.03	[−0.12, 0.02]	0.17
C2 * Emotional Support	**−0.18**	**0.07**	**[−0.32, −0.04]**	**0.01**	−0.01	0.04	[−0.09, 0.07]	0.80
C3 * Emotional Support	**−0.14**	**0.07**	**[−0.28, −0.02]**	**0.03**	−0.04	0.04	[−0.12, 0.03]	0.26
C4 * Emotional Support	−0.05	0.06	[−0.17, 0.07]	0.41	**−0.07**	**0.04**	**[−0.14, −0.00]**	**0.03**
Adjusted *R* ^*2*^	0.18				0.27			

*Note:* Statistically significant effects are bolded. Covariates: relationship status, chronic life burden.

Abbreviation: CI, confidence interval.

**FIGURE 2:**
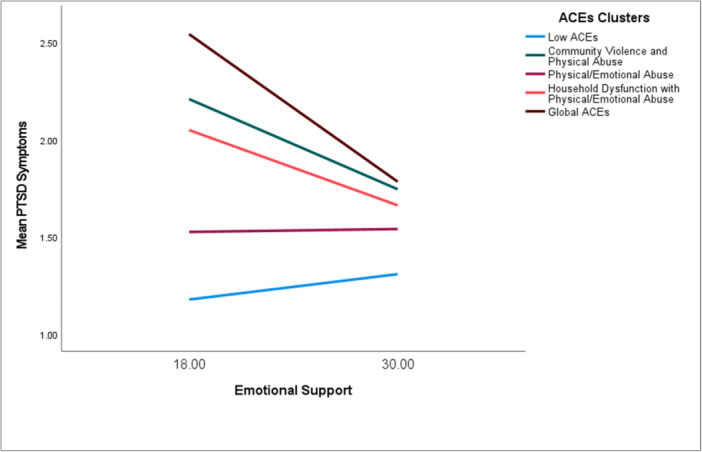
Post‐traumatic stress disorder symptoms across ACE clusters by emotional support level among Latina immigrants. *Note:* Emotional support total scores range from 18 to 30, low to high, respectively.

Women in the Community Violence and Physical Abuse and the Global ACEs clusters reported a greater burden of PTSD symptoms than women in the Low ACEs cluster. In examining the role of emotional support, moderation analyses showed a significant finding for the Global ACEs cluster (*b* = −0.07, SE = 0.04, *t* = −2.09, *p* = 0.037). This result suggests that the otherwise beneficial, buffering effect of emotional support on PTSD symptoms was significantly diminished or less effective for individuals in the Global ACEs cluster relative to the effect of emotional support observed in the Low ACEs cluster (Figure 2). Emotional support did not moderate the association between ACE clusters and symptoms of depression and anxiety (data not shown).

### 7.4 Mediating Effects of Emotional Support

We then examined whether emotional support mediated the association between ACEs clusters and mental health (depression and anxiety symptoms). We conducted two separate mediation models for each mental health outcome, which are presented in Table 4. Greater levels of emotional support were negatively associated with anxiety and depression symptoms (*Path b*). There were significant direct effects between ACE clusters and mental health (*Path c’*). The path between ACE clusters and depression was mediated by emotional support for women in the Household Dysfunction with Physical/Emotional Abuse (*B* = 0.38, *SE* = 0.20, CI: 0.05–0.83) and Global ACEs clusters (*B* = 0.55, *SE* = 0.29, CI: 0.08–1.22). Similarly, emotional support mediated the association between ACE clusters and anxiety for women in the Household Dysfunction with Physical/Emotional Abuse (*B* = 0.40, *SE* = 0.22, CI: 0.05–0.91) and Global ACEs clusters (*B* = 0.59, *SE* = 0.29, CI: 0.09–1.26).

**TABLE 4:** Mediating role of emotional support in the association between ACE clusters and mental health.

Outcomes	ACEs Clusters & Mediator	Path a (ACEs Emotional Support)		Path b		Total effect (c)		Direct effect (c’)		Indirect effect (a*b)		95% CI	Adjusted *R* ^2^
Depression	Low ACEs (ref)	β	SE	β	SE	β	SE	β	SE	β	SE		
	Community Violence and Physical Abuse	−1.51	1.10			**2.08**	**0.87** *	**1.91**	**0.87** *	0.17	0.16	[−0.05, 0.54]	
	Physical/Emotional Abuse	−1.07	1.12			0.01	0.89	−0.11	0.88	0.12	0.12	[−0.09, 0.42]	
	Household Dysfunction with Physical/Emotional Abuse	**−3.37**	**1.21**			1.01	0.96	0.63	0.96	**0.38**	**0.20**	**[0.05, 0.83]**	
	Global ACEs	**−4.86**	**1.27**			**2.67**	**1.0** **	**2.11**	**1.02** *	**0.55**	**0.29**	**[0.08, 1.22]**	
	Emotional Support			**−0.11**	**0.04** **								0.23

Outcomes	ACEs Clusters & Mediator	Path a (ACEs Emotional Support)		Path b		Total effect (c)		Direct effect (c’)		Indirect effect (a*b)		95% CI	Adjusted *R* ^2^
Anxiety	Low ACEs (ref)												
	Community Violence and Physical Abuse					**2.16**	**0.92**	**1.97**	**0.92** *	0.18	0.16	[−0.06, 0.57]	
	Physical/Emotional Abuse					0.39	0.95	0.25	0.94	0.13	0.13	[−0.10, 0.43]	
	Household Dysfunction with Physical/Emotional Abuse					**3.11**	**1.02** **	**2.70**	**1.02**	**0.40**	**0.22**	**[0.05, 0.91]**	
	Global ACEs					**2.44**	**1.07** *	1.84	1.08	**0.59**	**0.29**	**[0.09, 1.26]**	
	Emotional Support			**−0.12**	**0.04** *								0.25

*Note:* Covariates: relationship status, chronic life burden. Bold values are statistical significant at *p*‐value < 0.01.

* *p* < 0.05

** *p* < 0.01.

## 8 Discussion

This study offers a crucial contribution by being among the first to investigate the differential impact of emotional support on the relationship between distinct ACE exposure patterns, social problem‐solving, and mental health outcomes specifically among Latina immigrant women. Our findings reveal that the influence of ACEs on mental health varied by trauma type when emotional support was low. Notably, women in the Global ACEs cluster, characterized by exposure to the broadest range of traumas, reported the highest levels of depression, anxiety, and PTSD symptoms, coupled with lower social problem‐solving skills and less emotional support. Consistent with the stress‐buffering model, our results demonstrate that high levels of emotional support significantly ameliorated the detrimental effects of ACEs on both social problem‐solving and mental health outcomes. This underscores robust emotional support as a potent protective resource, enabling Latina immigrant women to more effectively manage daily challenges and reduce psychosocial distress, irrespective of their adverse childhood experiences.

Our results revealed lower scores for social problem‐solving in the low ACEs group compared to the other clusters in which women reported more types of adversity, indicating a correspondence with greater endorsement of maladaptive or ineffective coping styles. We have not identified other studies that have explored this association to compare findings. We offer potential explanations for this finding that acknowledge that individuals respond to adversity in diverse ways, and not all adversity leads to positive outcomes. The finding that individuals in the Low ACEs group demonstrated lower social problem‐solving scores than other clusters may reflect the concept of “steeling effects” or adversity‐related growth, wherein moderate exposure to manageable stressors can foster adaptive skill development (Rutter 2012). According to social learning theory, individuals who navigate early life challenges may develop enhanced problem‐solving abilities through repeated practice with difficult situations, learning to identify resources (Hermans et al. 2025). This pattern may reflect a curvilinear relationship rather than a linear one—minimal adversity may provide insufficient opportunities to develop these skills, and severe adversity can overwhelm adaptive capacity and impair functioning. Another explanatory factor to consider may be that of positive childhood experiences which we did not assess as part of this study. A growing body of evidence shows that positive childhood experiences are associated with better mental health and greater social and emotional support in adulthood, even when accounting for adverse childhood experiences (Bethell et al. 2019). For some of our study participants, the types of childhood adversity they experienced could have been moderated by positive experiences and caring guardians. These relationships deserve further exploration.

Although emotional support was not a moderator, we found that emotional support is a mediator in the association between ACEs and symptoms of depression and anxiety for women in the Household Dysfunction with Physical/Emotional Abuse and Global ACEs clusters. For women in the Household Dysfunction with Physical/Emotional Abuse and Global ACEs clusters, emotional support attenuated the impact of ACEs on depression and anxiety symptoms. Our study's findings are consistent with the literature demonstrating that emotional support can serve as a mediator between ACEs and mental health outcomes (Fitzgerald and Gallus 2020; Su et al. 2022). Our study contributes novel insights by demonstrating that the mechanism through which emotional support operates varies by ACE cluster type, with emotional support functioning as a mediator for certain adversity patterns while acting as a protective moderator for household dysfunction and global ACE exposures.

Our analysis revealed a distinct subgroup of women, the Global ACEs cluster, who experienced more types of childhood adversity. This cluster included various forms of interpersonal violence (sexual, physical, and emotional abuse), household dysfunction, collective violence, and bullying. As anticipated, these women displayed heightened vulnerability to psychological distress, reported lower levels of emotional support and social problem‐solving skills, and had the highest mean mental health symptom scores across all clusters. This observation aligns with existing research demonstrating the detrimental impact of poly‐victimization and high ACE scores on mental health and substance use (Shin et al. 2018).

Multiple or sustained childhood traumas can have profound and enduring developmental consequences and lead to more severe psychological distress in adulthood, including complex PTSD (Cloitre et al. 2009). This vulnerability to psychological distress may be attributed, in part, to the high prevalence of interpersonal traumas within the Global ACEs cluster. Early childhood exposure to interpersonal violence, particularly when perpetrated by caregivers, can disrupt the development of trust, security, and attachment and leave lasting impacts on social development (Charuvastra and Cloitre 2008; Cloitre et al. 2009; Muldoon et al. 2019).

For Latina immigrants, stressors associated with immigration (e.g., family separation, shifting family dynamics, migration‐related trauma) can further disrupt attachment patterns and social support networks, potentially exacerbating social isolation and psychological distress (Barton et al. 2022; Crocker 2015; Liddell et al. 2021; Ornelas et al. 2020; Rios Casas et al. 2020). Our findings suggest that emotional support may play a crucial role in fostering resilience by mitigating the relational impact of trauma on attachment, potentially alleviating PTSD symptoms for women in the Global ACEs cluster.

This study provides nuanced insights into the complex interplay between ACEs, mental health, and protective factors within a sample of Latina immigrant women. While acknowledging inherent limitations, our findings contribute to a growing body of literature that emphasizes the need for differentiated approaches to ACEs assessment and intervention.

It is important to acknowledge the limitations imposed by the study's cross‐sectional design. Although we identified significant associations between ACEs, emotional support, social problem‐solving skills, and mental health outcomes, the cross‐sectional nature precludes definitive conclusions regarding causality. Longitudinal studies are essential to delineate the temporal relationships between these variables and to elucidate the mechanisms through which ACEs exert their influence on long‐term mental health trajectories.

Furthermore, the generalizability of our findings is constrained by the sampling strategy. Our convenience sample, drawn from a specific community within one city, may not fully represent the diverse experiences of Latina immigrants across different socioeconomic strata, levels of acculturation, and immigration histories. Future research should prioritize more representative sampling approaches to ensure that findings are applicable to a broader spectrum of Latina immigrant women.

Finally, the utilization of the PROMIS Emotional Support scale, while providing a valuable measure of perceived support, does not offer granular insights into the specific sources of that support. Investigating the differential impact of various support sources (e.g., familial, partner, community, professional) could further refine our understanding of the protective mechanisms at play.

Notwithstanding these limitations, this study has important implications for clinical practice and nursing research. For clinical practice, findings support implementing trauma‐informed care approaches for Latina immigrant populations, including routine ACE screening and culturally responsive interventions that strengthen social support networks. Healthcare providers should recognize that emotional support may function differently across ACE exposure patterns—requiring support‐building interventions for some women while others may benefit from integrated trauma‐focused care addressing disrupted support systems.

For nursing science, this research contributes empirical evidence for the mechanisms through which social support influences recovery from childhood trauma, expanding the theoretical foundation for trauma‐informed care. The study advances practice by identifying specific protective factors that can be systematically assessed and strengthened through nursing interventions, providing concrete targets for both primary prevention (family‐centered interventions that strengthen support systems) and secondary prevention (early identification and targeted support for survivors).

Future research should examine how emotional support's protective mechanisms evolve as immigrants adapt to new environments, test whether differential effects require tailored nursing approaches, and explore optimal timing for support interventions across the migration trajectory. By focusing on these targeted directions, future studies can advance culturally responsive, trauma‐informed healthcare practices that enhance resilience and mitigate long‐term trauma effects in this underserved population.

## Author Contributions

Laura Mata Lopez, MSN, APRN, and Owen Smith, BSN, contributed to the original draft of the manuscript and participated in critical revisions. Maria Jose Sanchez‐Roman, MD, MPH, and Lia Escobar‐Acosta, BA, were responsible for data collection in the field and contributed to manuscript review and editing. Lia Escobar‐Acosta also provided supervision and project administration. Carmen Alvarez, PhD, APRN, obtained funding for the study and subsequently led the conceptualization and design, developed the methodology, conducted formal analysis and data curation, and was responsible for visualization, supervision, project administration, and writing both the original draft and subsequent revisions.

## Consent

Participants in this study were primarily recruited from a clinical setting, with the support of primary care providers who granted permission for recruitment within their clinics. Many of the individuals who participated were caregivers of patients receiving care at these clinics. Although patients, service users, or caregivers were not involved in the design, analysis, or interpretation of the study, their participation was central to the conduct of the research. The collaboration with clinical staff facilitated respectful and effective recruitment of caregivers, whose perspectives were vital to the study's aims.

## Conflicts of Interest

The authors declare no conflicts of interest.

## Data Availability

The data that support the findings of this study are available on request from the corresponding author. The data are not publicly available due to privacy or ethical restrictions.
